# Alterations in Human Mitral Valve Mechanical Properties Secondary to Left Ventricular Remodeling: A Biaxial Mechanical Study

**DOI:** 10.3389/fcvm.2022.876006

**Published:** 2022-06-09

**Authors:** Paulien Vandemaele, Klaas Vander Linden, Sébastien Deferm, Ramadan Jashari, Filip Rega, Philippe Bertrand, Pieter Vandervoort, Jos Vander Sloten, Nele Famaey, Heleen Fehervary

**Affiliations:** ^1^Biomechanics Section, Department of Mechanical Engineering, KU Leuven, Leuven, Belgium; ^2^Cardiology, Hospital Oost-Limburg, Genk, Belgium; ^3^Faculty of Medicine and Life Sciences, Hasselt University, Hasselt, Belgium; ^4^European Homograft Bank, Clinic Saint-Jean, Brussels, Belgium; ^5^Cardiac Surgery, University Hospitals Leuven, Leuven, Belgium; ^6^FIBEr, KU Leuven, Leuven, Belgium

**Keywords:** human mitral valves, secondary mitral regurgitation, left ventricular remodeling, planar biaxial and uniaxial testing, heart valve biomechanics, constitutive modeling, nonlinear parameter identification

## Abstract

Secondary mitral regurgitation occurs when a left ventricular problem causes leaking of the mitral valve. The altered left ventricular geometry changes the orientation of the subvalvular apparatus, thereby affecting the mechanical stress on the mitral valve. This in turn leads to active remodeling of the mitral valve, in order to compensate for the ventricular remodeling. In this study, a biomechanical analysis was performed on eight human mitral valves with secondary mitral regurgitation and ten healthy human mitral valves to better understand this pathophysiology and its effect on the mechanical properties of these tissues. Samples were obtained from the anterior and posterior leaflet and used for planar biaxial mechanical experiments. Uniaxial experiments were performed on four groups of mitral valve chords: anterior basal, anterior marginal, posterior basal and posterior marginal chords. The mechanical response of the mitral valve leaflets was fitted to the May-Newman and Yin constitutive model, whereas the material parameters of the third order Ogden model were determined for the chord samples. Next, stiffnesses calculated at low and high stress levels were statistically analyzed. Leaflet samples with secondary mitral regurgitation showed a small thickness increase and a change in anisotropy index compared to healthy control valves. Diseased leaflets were more compliant circumferentially and stiffer radially, resulting in anisotropic samples with the radial direction being stiffest. In addition, chord samples were slightly thicker and less stiff at high stress in secondary mitral regurgitation, when grouped per leaflet type and insertion region. These results confirm mechanical alterations due to the pathophysiological valvular changes caused by left ventricular remodeling. It is important that these changes in mechanical behavior are incorporated into computational models of the mitral valve.

## 1. Introduction

The mitral valve (MV) apparatus is a complex structure ensuring unidirectional blood flow between the left atrium and left ventricle during diastole. It consists of an annulus, two valve leaflets, two papillary muscles, and multiple chords. Insufficient leaflet coaptation during systole leads to leakage of the mitral valve or mitral valve regurgitation (MR) (1). MR is the most frequent valvular disease, with an estimated prevalence of 2% in US adults, which increases with age (2).

MR is classified as either primary when organic mitral valve disease is to blame or secondary in the setting of left ventricular disease. Secondary MR is often observed in patients with cardiomyopathy (CMP). The left ventricular remodeling causes papillary muscle displacement, which tethers the mitral valve leaflets and restricts normal leaflet closure (3).

Although this definition suggests a structurally normal mitral valve apparatus in secondary MR, studies show leaflets and chords actively adapt to this ventricular remodeling. Rausch et al. (4) showed with a chronic infarct ovine model that leaflet area can grow in both circumferential and radial direction due to chronic leaflet stretch as a result of papillary muscle tethering and annular dilation. Also Dal-Bianco et al. (5) reported active valve remodeling in response to mechanical stresses: larger and thicker MV leaflets and chords were observed in sheep after leaflet tethering. Further, Grande-Allen et al. (6) found thicker and longer MV leaflets with a higher concentration of collagen, glycosaminoglycans and cells and a lower water concentration in MR valves compared to healthy human valves. These compensatory mechanisms are however two-fold. Leaflet tethering stimulates leaflet growth, facilitating leaflet coaptation, but it also stimulates counterproductive thickening, thereby further impairing leaflet coaptation. A better understanding of these disease mechanisms is required to provide adequate treatment strategies.

Numerical modeling of the mitral valve apparatus is a helpful tool to investigate the mechanics of secondary MR and to improve the understanding of the disease mechanisms. This requires an accurate behavior description of both healthy and diseased mitral valves. Multiple studies reported biaxially derived properties of healthy animal (7–11) and human (12, 13) mitral valve leaflets, whereas healthy human chord properties were determined by Zuo et al. (14). Mechanical properties of human mitral valves with secondary MR on the other hand were determined by Grande-Allen et al. (15) and Prot et al. (16) based on uniaxial experiments of leaflets and chords. Further, Howsmon et al. (17) investigated the biaxial properties of ovine anterior leaflets with secondary MR.

Although several studies have tried to capture the mitral valve mechanical behavior, material parameters of human mitral valve leaflets with secondary MR derived from biaxial testing are still lacking. Biaxial testing of leaflets is required to capture their anisotropy for more reliable results. Therefore, this article presents an in-depth biomechanical characterization of healthy human mitral valves and mitral valves with secondary MR, using planar biaxial testing of leaflets and uniaxial testing of chords. The following sections explain the sample preparation and mechanical characterization process, then the results are presented and discussed.

## 2. Materials and Methods

### 2.1. Harvesting

Eighteen human mitral valves were collected from multi organ donors and heart transplant recipients from the European Homograft Bank: eight valves originating from patients with cardiomyopathy (CMP) showing mitral regurgitation (MR) and ten healthy control (HC) valves. Only patients without known medical history affecting the microstructure and mechanical properties of the valves were included. The mean age of the HC and MR group was 52.80±9.33 years and 51.88±11.19 years, respectively. Clinical details of the valve donors are presented in Table 1. The use of human tissue was approved by Comité d'Ethique Hospitalo-Facultaire Saint-Luc-UCL (CEHF).

**Table 1:** Details of the mitral valve donors, including weight, age, sex (female/male), and heart related disease.

**Specimen**	**Weight**	**Age**	**Sex**	**Heart related disease**
	**[kg]**	**[years]**	**[F/M]**	
HC 1	65	44	M	–
HC 2	63	53	F	–
HC 3	80	51	M	–
HC 4	63	54	M	–
HC 5	60	34	M	–
HC 6	95	52	M	–
HC 7	64	51	M	–
HC 8	50	67	F	–
HC 9	80	59	M	–
HC 10	77	63	F	–
MR 1	67	58	F	Non-ischemic dilated CMP
MR 2	83	53	F	Ischemic CMP
MR 3	90	59	M	Ischemic CMP
MR 4	105	69	M	Ischemic CMP
MR 6	75	31	F	Non-ischemic dilated CMP
MR 7	75	47	M	Non-ischemic dilated CMP
MR 9	80	52	M	Non-ischemic dilated CMP
MR 10	97	46	M	Ischemic CMP

After harvesting, the valves were transferred to the European Homograft Bank in saline (0.9%) at 4°C. After dissection and morphological evaluation, they were decontaminated with a cocktail of three antibiotics (Lincocin, Vancocin, and Polymyxin B) in 250mL of RPMI 1640 (Roswell Park Memorial Institute, Buffalo, New York, USA) for 20–48 h, followed by controlled cryopreservation in Planer 560-16 (Planer LTD, Sunbury-On-Thames, UK), using 10% dimethyl sulfoxide (DMSO) (WAK-Chemie Medical GmbH, Steinbach, Germany) as a cryoprotecting medium. Subsequently, the valves were stored in the vapors of liquid nitrogen at −179°C and transferred to the lab in a Dry Shipper (below −135°C), where they were stored at −80°C until their use for the experiments.

### 2.2. Sample Preparation

The mitral valves were thawed before testing according to the protocol of the European Homograft Bank by immersing the pouch consecutively in water and saline at a temperature of 37°C. The DMSO was diluted by rinsing the homografts with saline, decreasing progressively its concentration from 10% to 0% in four steps.

**Figure 1:**
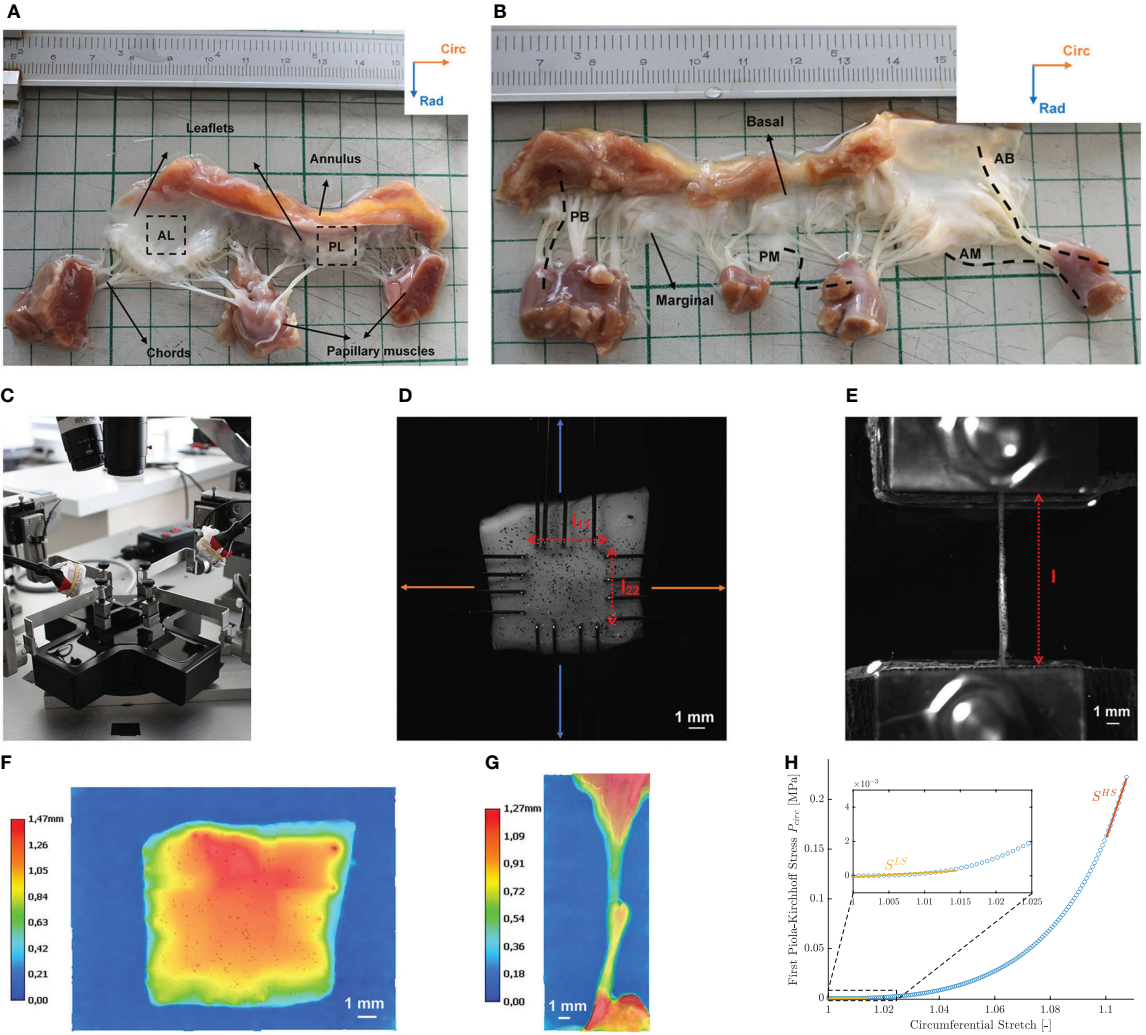
Overview of the different steps in the mechanical experiments on human mitral valves. **(A)** Square samples were excised from the anterior (AL) and posterior (PL) leaflet of a human MV with the edges aligned to the circumferential and radial direction. **(B)** Four types of chord samples were tested: anterior basal (AB), anterior marginal (AM), posterior basal (PB), and posterior marginal (PM) chords. **(C)** ZwickRoell planar biaxial tester used for the experiments. Samples were immersed in saline at a temperature of 37°C. **(D)** Leaflet samples were mounted in the device with the edges aligned to the circumferential and radial directions using rakes. *l*_11_ and *l*_22_ represent the distance between the opposing rakes in the circumferential and radial direction, respectively. **(E)** Mounted chord sample. Clamps were lined with sandpaper to prevent slipping of the samples. The distance between the clamps is indicated by *l*. **(F)** Thickness map of a leaflet sample obtained by 3D image stitching. **(G)** Diameter distribution of a chord sample obtained by 3D image stitching. Data of the annulus and papillary muscle were removed in the diameter analysis. **(H)** Linearized stiffness moduli were calculated as slope of the first Piola-Kirchhoff model stress at both low (LS) and high (HS) stress for each sample.

After thawing, square samples of 10mm × 10mm were excised from the mitral valve anterior (AL) and posterior (PL) leaflet for planar biaxial testing as seen in Figure 1A. Samples were chosen from the middle region of the leaflet with the edges along the circumferential, i.e., parallel to the annulus, and radial, i.e., perpendicular to the annulus, directions. A graphite powder speckle pattern was applied to the atrial surface of the sample to calculate its deformation during testing and a marker was attached to the top right corner to track the sample orientation.

After leaflet sample preparation, four chord samples were excised from each mitral valve for uniaxial testing as shown in Figure 1B. The mitral valve chords were categorized according to their leaflet type, anterior (A) or posterior (P), and insertion region, basal (B), or marginal (M). Basal chords insert close to the annulus, whereas marginal chords insert at the tip of the leaflet. Parts of the leaflet or annulus and papillary muscle were included in the chord samples to facilitate clamping.

All tissue samples were conserved in saline at a temperature of 4°C prior to testing and were tested within 10h after thawing.

### 2.3. Thickness Measurement

A height map of each leaflet and chord sample was obtained by 3D image stitching with a Keyence VHX 6000 3D Digital Microscope (Keyence Corporation, Osaka, Japan) (magnification ×50) as shown in Figures 1F,G, respectively. After processing in Matlab2019b (The Mathworks Inc., Natick, Massachusetts, USA), the mean value of the height map and its standard deviation were obtained, representing the mean thickness or mean diameter of a leaflet and chord sample, respectively, and the thickness or diameter variation within the sample.

### 2.4. Mechanical Testing

#### 2.4.1. Planar Biaxial Testing

The leaflet samples were mounted in a ZwickRoell planar biaxial tester (ZwickRoell Testing Systems GmbH, Fürstenfeld, Austria) using four sets of rakes as shown in Figures 1C,D. Each rake consisted of four needles with diameter 0.3mm, spacing 1.25mm and puncture depth of 1mm. The circumferential and radial direction of the samples were aligned with the two test axes of the ZwickRoell tester. The samples were loaded with a displacement-controlled protocol and the resulting forces in the sample were measured by two load cells on each axis at a sample rate of 20Hz. The sample deformation was captured by a G917 Manta camera (Allied Vision, Stadtroda, Germany) mounted perpendicularly to the sample at a frequency of 20Hz. All samples were immersed in saline at a temperature of 37°C during testing.

**Figure 2:**
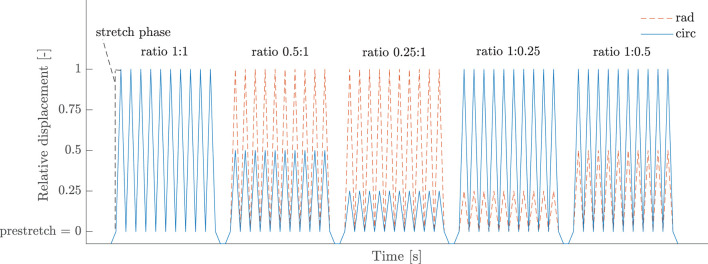
Displacement-controlled protocol of a biaxial test. This part consists of five loading ratios with each ten preconditioning cycles and is repeated for several strain levels. The relative displacement in both circumferential and radial direction is defined with respect to the position at which preload has been reached and is given as a fraction of the imposed strain level.

The test protocol consisted of different loading cycles, consisting of a stretch and recover phase and determined by a strain level and ratio *circ:rad* by which the strain level was applied in circumferential and radial direction. Samples were stretched in both directions at a speed of $0.1\frac{\text{mm}}{\text{s}}$ until a preload of 0.01N was reached to avoid sagging of the sample. Next, the actuators moved at a strain rate of $2.5\frac{\%}{\text{s}}$ to the predefined strain level in the stretch phase and moved back to their preload position in the recovery phase. This loading cycle was repeated ten times to take care of the hysteresis between loading and unloading, also referred to as preconditioning. Only the highest reached stretch phase was used in further analysis. An overview of the applied strain levels and ratios in the biaxial test protocol is given in Table 2 and the biaxial test protocol is visualized for one strain level in Figure 2.

**Table 2:** Overview of the different strain levels, ratios, and preconditioning cycles in the biaxial and uniaxial test protocol.

	**Biaxial test**	**Uniaxial test**
Strain levels (%)	2.5, 5, 7.5, 10, 15, 20	2.5, 5, 7.5, 10, 15, 20,
		25, 30, 35, 40, 45, 50
Ratios (circ:rad)	1:1, 0.5:1, 0.25:1, 1:0.25, 1:0.5	–
Preconditioning cycles	10	10

#### 2.4.2. Uniaxial Testing

The chord samples were mounted in a ZwickRoell planar biaxial tester using clamps with manual control, as shown in Figure 1E. The clamps were lined with sandpaper to avoid slipping of the samples. Samples were immersed in saline at a temperature of 37°C and loaded with a displacement-controlled protocol. The test protocol consisted of ten preconditioning cycles and twelve different strain levels as presented in Table 2. Samples were stretched at a speed of $0.5\frac{\text{mm}}{\text{s}}$ until a preload of 0.03N was reached to avoid sagging of the sample. In the stretch phase, the actuators moved at a strain rate of $5\frac{\%}{\text{s}}$ to the predefined strain level after which they moved back to their preload position in the recovery phase. The stretch part of the last cycle was used in further analysis.

### 2.5. Constitutive Models

The stretch phase of the tenth loading cycle with the highest reached strain level was used as an input for constitutive modeling. Both the leaflets and the chords were considered incompressible. The constitutive models discussed in this section are therefore isochoric.

#### 2.5.1. Leaflets

The mechanical response of the mitral valve leaflets was fitted to the May-Newman and Yin (MN) constitutive model (18). This hyperelastic, incompressible and transversely isotropic material model is given by the strain energy density function Ψ in Equation (1).


(1) $$\begin{array}{l} \text{Ψ}=c_{10}\left[\exp\left(c_{1}{\left(I_{1}-3\right)}^{2}+c_{2}{\left(\sqrt{I_{4}}-1\right)}^{4}\right)-1\right] \end{array}$$

The right Cauchy Green tensor ***C*** is given by the deformation gradient tensor ***F***, ***C*** = ***F***^*T*^***F***. The first invariant *I*_1_ of the right Cauchy Green tensor ***C*** depends on the principal stretches λ_*i*_,


$$I_{1}=trC=\lambda_{1}^{2}+\lambda_{2}^{2}+\lambda_{3}^{2},$$

and the fourth pseudoinvariant *I*_4_ of the right Cauchy Green tensor ***C*** depends on the fiber angle α w.r.t. the circumferential direction,


$$I_{4}=a^{0}\cdot \left(Ca^{0}\right),$$

with


$$a^{0}=\left[\begin{array}{c} \cos\alpha \\ \sin\alpha \\ 0 \end{array}\right].$$

The order of the axes is given as circumferential, radial and axial. The unknown material parameters *c*_10_, *c*_1_, and *c*_2_ and structural parameter α were determined based on the experimental results.

#### 2.5.2. Chords

The stress-stretch curves of the mitral valve chords were fitted to the third order Ogden model for incompressible, hyperelastic, isotropic materials as given by Equation (2) (19). This constitutive model is expressed in the principal stretches λ_1_, λ_2_, λ_3_ and has six unknown parameters μ_*i*_ and *a*_*i*_ (*i* = 1, 2, 3).


(2) $$\begin{array}{l} \text{Ψ}=\overset{3}{\underset{i=1}{\sum }}\frac{\mu_{i}}{a_{i}^{2}}\left(\lambda_{1}^{a_{i}}+\lambda_{2}^{a_{i}}+\lambda_{3}^{a_{i}}-3\right) \end{array}$$

The shear modulus μ is given by $2\mu =\overset{3}{\underset{i=1}{\sum }}\mu_{i}a_{i}$ in the undeformed stress-free configuration. Hence, for a physically realistic response and material stability μ_*i*_*a*_*i*_ > 0, for *i* = 1, 2, 3 (20).

### 2.6. Parameter Fitting

The constitutive model parameters were determined minimizing the difference between the model and experimental reaction forces ***RF*** in the experimental test directions according to objective function


(3) $$\begin{array}{l} \frac{1}{n}\underset{ii}{\sum }{\left[\left({RF}_{ii}^{mod}-{RF}_{ii}^{exp}\right)\times 100\right]}^{2}, \end{array}$$

with *n* the number of data points and *ii* the directions of the test, i.e., 11 and 22 for planar biaxial and 22 for uniaxial.

The experimental reaction forces ***RF***^*exp*^ were calculated as the average of the measured forces by the two load cells on the circumferential and radial axes for the biaxial experiments and as the average of the measured forces by the two load cells on the axial axes for the uniaxial experiments. For some samples, the measurements of only one of the load cells were used due to force recording problems. Data of the tenth stretch phase of ratio 1:1 of the highest reached loading cycle were used. The first data point of this loading cycle was set as the reference point for the undeformed state.

The model reaction forces ***RF***^*mod*^ were derived from the deformation gradient tensor and the constitutive model. First, the model second Piola-Kirchhoff stress ***S***^*mod*^ was calculated from the strain energy density function, $S=2\frac{\partial \text{Ψ}}{\partial C}-pC^{-1}$. The Lagrange multiplier *p* was determined such that σ_33_ = 0 with the Cauchy stress **σ** = *J*^−1^***FSF***^*T*^. The first Piola-Kirchhoff stress tensor ***P*** was calculated as ***P*** = ***FS***. The model reaction forces ***RF***^*mod*^ were then finally obtained by multiplying the first Piola-Kirchhoff stress tensor ***P*** with the cross-sectional area of the undeformed sample ***A***^*u*^, ***RF***^*mod*^ = ***PA***^*u*^. In the paper, superscripts *u*, *p* and *s* refer to dimensions in the unloaded, preloaded and loaded configuration, respectively. For a more detailed description of the different configurations, the reader is referred to (21).

Since the reference point was set at the point preload was reached, the reference configuration was not completely stress-free. In order to find the stress-free configuration, the deformation gradient tensor ***F*** was decomposed multiplicatively into a preload part ***F_p_*** and a stretch part ***F_s_***, ***F*** = ***F_s_*** · ***F_p_***.

The stretch part ${F_{s}}^{leaflet}$ of the deformation gradient tensor for the biaxial experiments on the leaflets was calculated based on digital image correlation (DIC) measurements on the atrial surface of the sample and is given by Equation (4). λ_11_ and λ_22_ are the stretches in the circumferential and radial direction, respectively, whereas λ_12_ and λ_21_ are shear stretches. All of them were obtained from the DIC strainmap using the Vic-2D 6 software (Correlated Solutions, Inc., South Carolina, US), integrated by isi-sys (isi-sys GmbH, Kassel, Germany). Incremental correlation was used with step size 7 and subset size 41. At each time point the mean stretch of the inner 50% central area enclosed by the rakes was used. λ_33_ was then determined assuming incompressibility of the tissue: $\det{F_{s}}^{leaflet}=J=1$.


(4) $$\begin{array}{l} {F_{s}}^{leaflet}=\left(\begin{array}{ccc} \lambda_{11} & \lambda_{12} & 0\\ \lambda_{21} & \lambda_{22} & 0\\ 0 & 0 & \frac{1}{\lambda_{11}\lambda_{22}-\lambda_{12}\lambda_{21}} \end{array}\right) \end{array}$$

The stretch part ${F_{s}}^{chord}$ of the deformation gradient tensor for the uniaxial tensile tests on the chords is given in Equation (5) and was calculated based on the displacement of the clamps. No DIC measurement or marker tracking was possible for these samples due to the small sample width. The stretch along the experimental test direction is given by the ratio of the distance between the clamps during the test *l*^*s*^ and the initial distance between the clamps at preload position *l*^*p*^ as indicated in Figure 1E, $\lambda_{22}=\frac{l^{s}}{l^{p}}$. λ_11_ and λ_33_ are assumed equal and calculated based on the incompressibility condition $\det{F_{s}}^{chord}=J=1$.


(5) $$\begin{array}{l} {F_{s}}^{chord}=\left(\begin{array}{ccc} \frac{1}{\sqrt{\lambda_{22}}} & 0 & 0\\ 0 & \lambda_{22} & 0\\ 0 & 0 & \frac{1}{\sqrt{\lambda_{22}}} \end{array}\right) \end{array}$$

The preload part ${F_{p}}^{leaflet}$ of the deformation gradient tensor is given in Equation (6) for the mitral valve leaflets. $G_{11}^{leaflet}$ and $G_{22}^{leaflet}$ are the prestretches in the circumferential and radial direction, respectively. $G_{33}^{leaflet}$ is calculated based on the incompressibility assumption $\det{F_{p}}^{leaflet}=1$.


(6) $$\begin{array}{l} {F_{p}}^{leaflet}=\left(\begin{array}{ccc} G_{11}^{leaflet} & 0 & 0\\ 0 & G_{22}^{leaflet} & 0\\ 0 & 0 & \frac{1}{G_{11}^{leaflet}G_{22}^{leaflet}} \end{array}\right) \end{array}$$

Equation (7) shows the preload part ${F_{p}}^{chord}$ of the deformation gradient tensor for the uniaxial tests on the chords. $G_{11}^{chord}$ and $G_{33}^{chord}$ are assumed to be equal and are determined to meet the incompressibility condition.


(7) $$\begin{array}{l} {F_{p}}^{chord}=\left(\begin{array}{ccc} \frac{1}{\sqrt{G_{22}^{chord}}} & 0 & 0\\ 0 & G_{22}^{chord} & 0\\ 0 & 0 & \frac{1}{\sqrt{G_{22}^{chord}}} \end{array}\right) \end{array}$$

These prestretches $G_{11}^{leaflet}$, $G_{22}^{leaflet}$ and $G_{22}^{chord}$ are unknown and are optimization variables determined in the parameter fitting as explained at the end of this section.

The initial cross-sectional area of the leaflets ***A***^*u,leaflet*^ was found by multiplying the measured sample thickness *t*^*u*^ with the distance between the opposing rakes in the unloaded configuration *l*^*u*^ as depicted in Figure 1D. For this, the measured distances at the moment of preload $l_{11}^{p}$ and $l_{22}^{p}$ needed to be corrected with the prestretches $G_{11}^{leaflet}$ and $G_{22}^{leaflet}$. The resulting undeformed cross-sectional area of the leaflets is given in Equation (8). The cross-sectional area of the chord samples was calculated based on the measured diameter *d*^*u*^, $A^{u,chord}=\frac{{\left(d^{u}\right)}^{2}\pi }{4}$.


$$\begin{array}{lll} A^{u,leaflet} & = & \left(\begin{array}{ccc} t^{u}l_{22}^{u} & 0 & 0\\ 0 & t^{u}l_{11}^{u} & 0\\ 0 & 0 & l_{11}^{u}l_{22}^{u} \end{array}\right) \end{array}$$

(8) $$\begin{array}{ll} = & \left(\begin{array}{ccc} t^{u}\frac{l_{22}^{p}}{G_{22}^{leaflet}} & 0 & 0\\ 0 & t^{u}\frac{l_{11}^{p}}{G_{11}^{leaflet}} & 0\\ 0 & 0 & \frac{l_{11}^{p}}{G_{11}^{leaflet}}\frac{l_{22}^{p}}{G_{22}^{leaflet}} \end{array}\right) \end{array}$$

All calculations were performed in Matlab2019b. The objective function given in Equation (3) was minimized using CasADi (22), an open-source tool for nonlinear optimization, taking into account the constraint of Equation (2) for the chord samples. To avoid ending up in a local minimum, 10 different sets of initial parameters were used. The parameter boundaries used during the fitting of the leaflet and chord samples are presented in Table 3. Due to the large difference in parameter boundaries, the parameters of the MN model were scaled between 0 and 1 to enhance numerical optimization.

**Table 3:** Upper and lower boundaries of the MN and third order Ogden constitutive model parameters used in the parameter fitting.

	**MN**				**Ogden**	
	***c*_10 [MPa]**	***c*_1 [–]**	***c*_2 [–]**	**α [rad]**	**μ_i_ [MPa]**	***a*_i_ [–]**
Lower boundaries	10^−9^	10^−4^	10^−4^	$\frac{-\pi }{2}$	−250	−250
Upper boundaries	1	500	5, 000	$\frac{\pi }{2}$	250	250

The goodness of fit was evaluated using the normalized root mean square error (NRMSE) of the experimental and model reaction forces as given in Equation (9). The error is normalized as it scales with the observed range of experimental reaction forces.


(9) $$\begin{array}{c} NRMSE=\frac{\sqrt{\frac{1}{n}\sum_{ii}{\left[(RF_{ii}^{mod}-RF_{ii}^{exp})\times 100\right]}^{2}}}{mean_{ii}(\text{max}RF_{ii}^{exp}-\text{min}RF_{ii}^{exp})} \end{array}$$

To determine the unknown prestretch values $G_{11}^{leaflet}$ and $G_{22}^{leaflet}$ for the leaflets, the optimization procedure was performed for all prestretch values $G_{ii}^{leaflet}$ from 1 to 1.1 with increments of 0.01. The prestretch and material parameters with the lowest NRMSE were then selected as optimized solution. Prestretch $G_{22}^{chord}$ of the chord samples was determined alike, with $G_{22}^{chord}$ ranging from 1 to 1.05 with increments of 0.001.

### 2.7. Data Analysis: Stiffness Moduli and Anisotropy Index

Linearized stiffness moduli were calculated as slope of the first Piola-Kirchhoff model stress as a function of stretch. Stress-stretch curves were generated in the experimental stress range based on the calibrated material parameters and an equibiaxial deformation gradient. The slopes of the stress-stretch curves of the chords at low (LS) and high stress (HS) were determined by fitting the first and last 21 data points with a first order polynomial. As shown in Figure 1H, only 11 data points were used to calculate the slope at high stress for the mitral valve leaflets due to the high nonlinearity of the MN model. To investigate the anisotropy of the mitral valve leaflets, an anisotropy index (AI) was defined as the ratio of the stiffness in circumferential and stiffness in radial direction for both low and high stress. All data processing was performed in Matlab2019b.

### 2.8. Statistical Analysis

Standard statistical methods were used to analyze the mechanical data. Data were first tested for normality using the Lilliefors test at the 1% significance level. The *p*-value was calculated with a Monte Carlo simulation with a maximum Monte Carlo standard error of 0.001. Two-sample *t*-tests with (un)equal variances were performed for normal distributed data to compare the means between groups. A paired *t*-test was performed when data were compared from the same sample. Equality of the variances was tested on beforehand with the *F*-test for normal distributed data at the 1% significance level. When data were not normally distributed, a two-tailed Wilcoxon rank sum test or Wilcoxon signed rank test for paired samples was used to compare the medians of different groups. A Bonferroni correction was added to correct for the multiple comparisons and hence to control the family-wise error rate. A *p*-value less than 0.05 was considered statistically significant with *p* < 0.01 highly significant. All statistical analysis was performed in Matlab2019b.

## 3. Results

**Figure 3:**
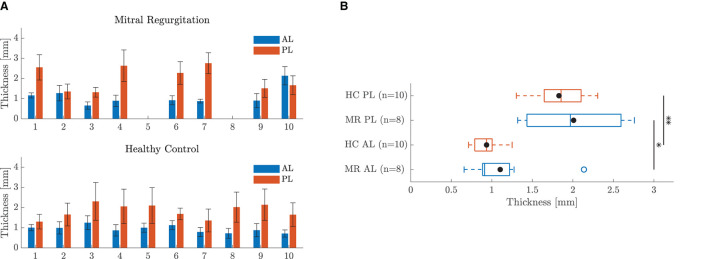
Thickness of the leaflet samples. **(A)** Bar chart of the thickness map of each leaflet sample: anterior leaflet (AL), posterior leaflet (PL). Data are represented as mean ± standard deviation of the height map obtained by the Keyence microscope. **(B)** Boxplot of the mean sample thickness of all leaflet samples per group. The mean value of each group is marked with •. *Indicates a statistically significant difference (*p* < 0.05) and ** a highly statistically significant difference (*p* < 0.01).

**Figure 4:**
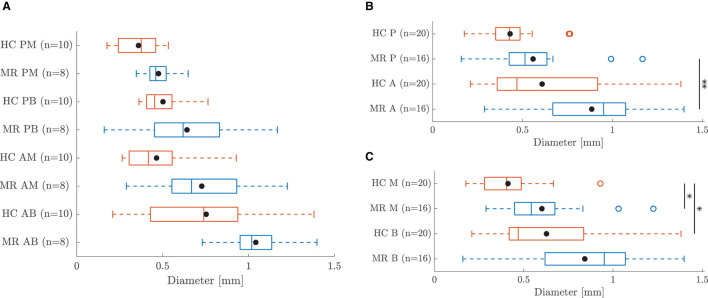
Boxplot of the diameter of all chord samples grouped **(A)** per chordal type **(B)** per leaflet type and **(C)** per insertion region. The mean value of each group is marked with •. *Indicates a statistically significant difference (*p* < 0.05) and ** a highly statistically significant difference (*p* < 0.01).

The thickness of each leaflet sample is shown in Figure 3A. Data are represented as mean ± standard deviation of the height map obtained by the Keyence microscope. The standard deviation is a measure of the heterogeneity of the sample. The average sample thicknesses are also visualized in the boxplots of Figure 3B for each leaflet group. The diameters of the different chord samples are shown in Figure 4A for each chordal type and were further grouped per leaflet type in Figure 4B and per insertion region in Figure 4C. Data were checked for normality prior to testing. All data groups were normally distributed with the exception of the HC basal chord samples. Due to the large sample size of this group (*n* = 20), normal distribution was assumed and a two-sample *t*-test was performed for each category. The mean value and standard deviation of the leaflet thickness and chord diameter per group are given in Supplementary Tables 1, 4, respectively.

**Figure 5:**
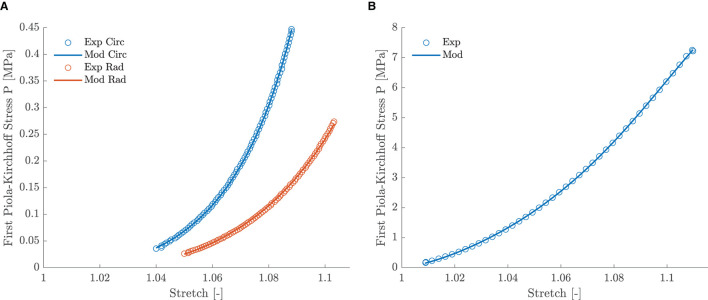
**(A)** Experimental and model stress-stretch curves of leaflet sample HC 9 AL with a NRMSE of 0.50. **(B)** Experimental and model stress-stretch curves of chord sample MR 9 AM with a NRMSE of 0.21.

**Figure 6:**
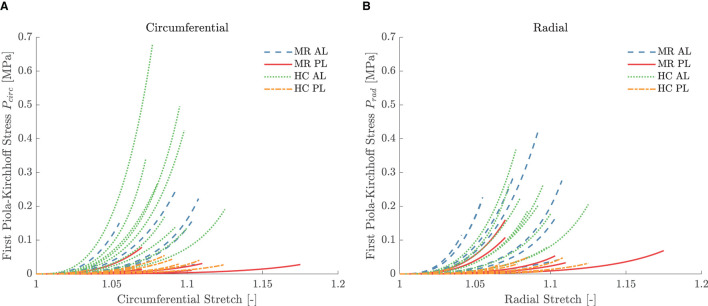
Equibiaxial first Piola-Kirchhoff stress versus stretch in the **(A)** circumferential and **(B)** radial direction of the different leaflet samples. The model stress was derived by parameter fitting to the MN constitutive model. The stretch range was defined based on the experimental data of the highest reached strain level for each sample.

**Figure 7:**
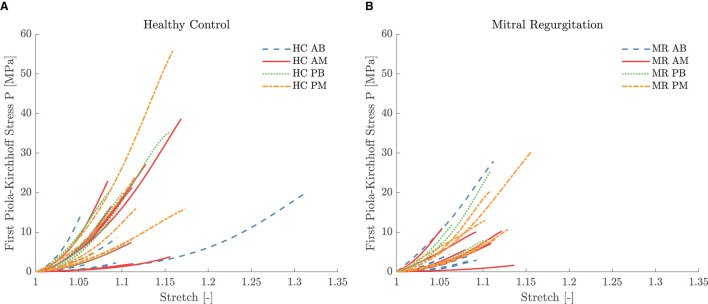
First Piola-Kirchhoff stress versus stretch per chordal group for the **(A)** HC and **(B)** MR valves. The model stress was derived by parameter fitting to the third order Ogden constitutive model. The stretch range was defined based on the experimental data of the highest reached strain level for each sample.

**Figure 8:**
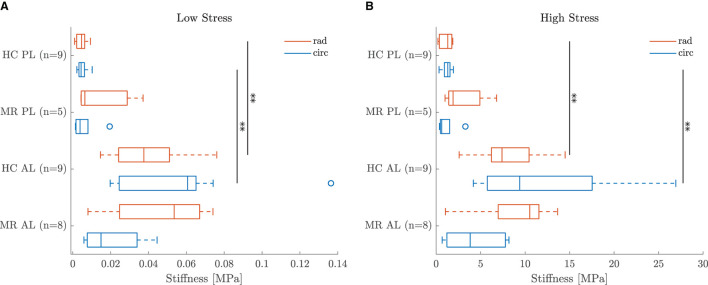
Boxplot of the leaflet sample stiffness at **(A)** low and **(B)** high stress per group. **Indicates a highly statistically significant difference (*p* < 0.01).

**Figure 9:**
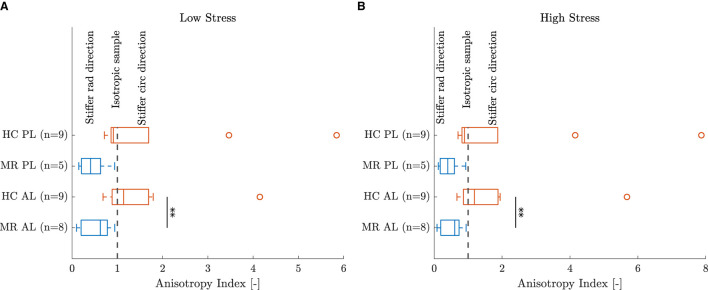
Anisotropy index of the leaflet samples at **(A)** low and **(B)** high stress. **Indicates a highly statistically significant difference (*p* < 0.01).

**Figure 10:**
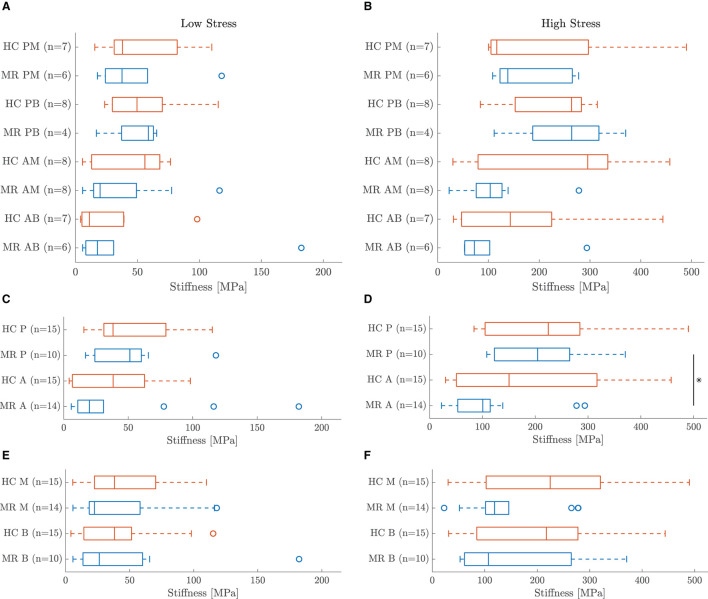
Boxplots of the chord sample stiffness at **(A,C,E)** low and **(B,D,F)** high stress. Chords are grouped **(A,B)** per chordal type, **(C,D)** per leaflet type and **(E,F)** per insertion region. *Indicates a statistically significant difference (*p* < 0.05).

Five leaflet samples and eighteen chord samples were excluded from the mechanical analysis due to an insufficient amount of collected data, problems during force capturing, slipping of the sample or a poor fitting (visual interpretation). A visualization of the fitting to the experimental data is given in Figure 5 for a representative leaflet and chord sample. The model stress-stretch curves resulting from the parameter fitting are shown in Figures 6, 7 for the mitral valve leaflets and chords, respectively. Linearized stiffness moduli at both low and high stress were calculated from these curves and these boxplots are shown in Figures 8, **10** for each leaflet and chord group, respectively. The stiffness of the chord samples was also grouped per leaflet type and per insertion region. The AI of the leaflet samples at low and high stress is shown in Figure 9. A Wilcoxon rank sum test or Wilcoxon signed rank test was used to compare the stiffness and AI of different groups due to the different sample sizes. Statistically significant groups were indicated with * (*p* < 0.05) and high significance was indicated with ** (*p* < 0.01). The data supporting the boxplots, i.e., median ± interquartile range of the stiffness and AI are available in Supplementary Tables 2, 3, 5. A summary of the results for each leaflet and chord sample is available in Supplementary Tables 6–11.

## 4. Discussion

Both healthy and diseased human mitral valves were mechanically characterized in this study to provide a thorough understanding of the adaptive changes seen in mitral valve leaflets and chords following left ventricular remodeling. The HC and MR valves originated from the same aged population, between 31 and 69 years old, making it possible to directly compare the results between the groups. Moreover, effects of aging can be neglected due to the relative young patient group. To our knowledge, this is the first time human mitral valve leaflets with secondary MR are tested biaxially and the first study to assess the changes in secondary MR for different chordal groups.

The analysis of the results is divided into four parts, discussing leaflet morphology, leaflet mechanics, chord morphology, and chord mechanics, respectively. Each time, regional valvular differences were analyzed first, after which a comparison was made between healthy and diseased valves. The discussion ends with the clinical relevance and limitations and future work of this study.

### 4.1. Leaflet Morphology

#### 4.1.1. Anterior vs. Posterior

All HC and almost all MR valves showed a thicker posterior than anterior leaflet sample as seen in Figure 3A. The thickness map variation was in general also larger for the posterior leaflet samples, suggesting this leaflet is less homogeneous in thickness. The mean sample thicknesses grouped per category are given in Figure 3B. The mean posterior leaflet thickness was significantly higher than the anterior leaflet thickness for both HC (*p* < 0.01) and MR valves (*p* < 0.05). Similar conclusions were found by Pham et al. (13) for aged healthy human mitral valve leaflets; showing posterior leaflets were significantly thicker than anterior leaflets, whereas other studies did not observe a thickness difference between the leaflets (11, 12, 15). Note that this difference could be related to the fact that the anterior leaflet thickness strongly depends on the region the sample was taken from. The anterior leaflet belly region is significantly thinner compared to the edge (12).

#### 4.1.2. Healthy vs. Diseased

Both anterior and posterior leaflet MR samples were slightly thicker than the HC samples as shown in the boxplot in Figure 3B, but these differences were not statistically significant. Different conclusions are reported in literature. Grande-Allen et al. (6) found significantly higher thicknesses in MR valves as opposed to HC valves when thickness was derived from 2D echocardiographic measurements. However, in another study the same group found that MR valves were thinner than HC valves, measured with a digital caliper this time (15). The reported thickness values were also higher than the ones found in our study. This could be due to the location from which the sample was taken and the measurement technique.

### 4.2. Leaflet Mechanics

#### 4.2.1. Anterior vs. Posterior

The MN constitutive model was capable of describing the nonlinear leaflet behavior as can be seen in Figure 5A. Although a large patient variability was observed in the mechanical leaflet response, the stress-stretch curves in Figure 6 show a clear difference between the anterior and posterior leaflet samples: the latter group was more compliant at low stress in both circumferential and radial direction compared to the anterior leaflet samples. This difference is quantified by the slopes of the stress-stretch curves at both low and high stress, as can be seen in Figure 8. The anterior leaflet samples were stiffer in circumferential and radial direction than the posterior leaflet samples for both HC (*p* < 0.01) and MR valves at both stress levels. These inter-leaflet differences were also observed in previous biaxial studies on human mitral valve leaflets (12, 13). Pham et al. (13) reported tangent moduli at high load of the anterior leaflet in the same range as our results, namely 12.82±3.96MPa and 6.89±2.26MPa for the circumferential and radial direction, respectively, and for the posterior leaflet 4.08±0.77MPa and 0.59±0.11MPa in the circumferential and radial direction, respectively. Previous studies on porcine mitral valve leaflets only observed significant differences in leaflet stiffness in the circumferential direction at low (11) and high load (7), respectively.

Further, Figure 8 also shows that the circumferential direction of the HC anterior leaflet samples was stiffer than the radial direction at both low and high stress, but this difference was not statistically significant. No directional difference could be observed for the HC posterior leaflet samples, suggesting the posterior leaflet samples to be more or less isotropic. This (an)isotropy is quantified for each sample separately by the AI showed in Figure 9 for both low and high stress. It can be seen that most of the HC posterior leaflet samples have an AI slightly smaller than one and hence these leaflets are close to isotropic with the radial direction being slightly stiffer than the circumferential direction. The HC anterior leaflet samples on the contrary, have an AI mainly larger than one, indicating the circumferential direction being the stiffest one. This difference in AI between the leaflet types is however not statistically significant. Previous biaxial studies on human and porcine mitral valve leaflets observed this anisotropic character with the circumferential direction being the stiffest one, in contrary to our results, for both anterior and posterior leaflets (7–13). Also May-Newman and Yin (7) found the posterior leaflet being more isotropic than the anterior leaflet, but no study reported a stiffer radial than circumferential direction in the posterior leaflet. Pham et al. (13) even found human anterior leaflets more isotropic than posterior leaflets: at high load, the circumferential response was two times higher than the radial response for the anterior leaflet, whereas it was seven times higher for the posterior leaflet.

The observed mechanical differences between anterior and posterior leaflet and the observed leaflet anisotropy can be explained by the valve's microstructure. Each leaflet contains a certain distribution of extracellular matrix proteins elastin and collagen, providing elasticity and strength to the tissue, respectively. Roberts et al. (23) investigated the distribution of collagen and elastin fibers in porcine mitral valve leaflets. Histological analysis showed a high concentration of circumferentially aligned collagen fibers in the anterior leaflet, whereas no or only few alignment was seen in the radial direction. The opposite was observed for the elastin fibers: the elastin fibers were predominantly aligned along the radial direction and there was little directionality in the circumferential direction. As a result, the circumferential direction of the anterior leaflet is the stiffest one, whereas the radial direction is more extensible. The posterior leaflet on the other hand showed a less pronounced directionality of the collagen and elastin fibers in the central region of the leaflet. Some circumferentially aligned collagen fibers were present in the posterior leaflet, but to a much lower extent than in the anterior leaflet.

These microstructural findings agree well with our median mechanical results: anterior leaflet samples are highly anisotropic with the circumferential direction being the stiffest one, whereas the anisotropy is much less pronounced in the posterior leaflet samples. However, the boxplot of the experimental results in Figure 9B also shows anterior leaflet samples with an AI smaller than one and posterior leaflet samples with an AI much larger than one with a stiffness in the circumferential direction four to eight times higher than in the radial direction. Pham et al. (12) also found anterior leaflets with a stiffer radial than circumferential direction, but this reverse anisotropy was attributed to the calcifications observed on the aged human mitral valves and therefore excluded from the study. The valves considered in our study are originating from much younger donors (between 31 and 69 years old) and most valves showed only minor calcifications around the annulus.

The large variation in AI might be due to the positioning of the sample on the valve. The posterior leaflet samples covered mainly the whole leaflet, whereas samples were taken from the central region for the anterior leaflets. Depending on the leaflet size, the distance to the annulus and edge might slightly differ for the different samples. Laurence et al. (8) investigated how the mechanical properties varied along the porcine mitral valve anterior leaflet and observed a higher anisotropy for the central regions compared to the edge regions.

#### 4.2.2. Healthy vs. Diseased

A shift is observed in the characteristic anisotropy for both anterior and posterior leaflets with secondary MR. Figure 9 shows that the AI at low and high stress of both anterior and posterior leaflet samples is lower than one for the MR group, indicating highly anisotropic samples with the radial direction being stiffer than the circumferential direction. Hence, the AI of the MR group is lower than the one of the HC group at both low and high stress and this difference is significant for the anterior leaflet samples (*p* < 0.01).

This difference in AI at both low and high stress is explained in Figure 8. A lower stiffness was observed in the circumferential direction of the MR anterior leaflet samples in contrast to the HC group, whereas a slightly higher stiffness was seen in the radial direction of the MR group compared to the HC group. As a result, the radial direction of the anterior leaflet is stiffer than the circumferential direction in the MR valves. The same trend was found for the posterior leaflet samples, but to a much lower extent.

Grande-Allen et al. (15) compared mitral valves of donors with dilated and ischemic CMP to healthy autopsy valves. In their study, the uniaxially tested anterior leaflet samples were significantly stiffer and had a lower extensibility in both circumferential and radial direction in the MR group compared to the HC group. An increase in stiffness in the MR group was also found for the circumferential direction of the posterior leaflet, but this difference was not significant. No radial samples were obtained from the HC posterior leaflets due to the small leaflet size and hence no comparison of the radial properties between MR and HC posterior leaflets could be made. Prot et al. (16) tested the anterior leaflet of a healthy human valve and a valve from a CMP heart uniaxially and found that the anterior leaflet from the CMP heart was more extensible in both directions than the HC valve. However, these results were both derived from uniaxial experiments on mitral valve leaflet strips in circumferential and radial direction, which is not representative for the *in vivo* biaxial loading situation. Furthermore, the tested circumferential and radial strips were each time originating from different valve leaflets due to the limited leaflet size. Previously, May-Newman and Yin (7) showed that porcine mitral valve leaflet properties derived from equibiaxial and strip biaxial tests did not differ significantly in the circumferential direction, but significant differences were observed in the radial direction. Hence, the interaction during loading between both directions cannot be neglected, which confirms the importance of planar biaxial testing over uniaxial testing.

Biaxial mechanical experiments were performed by Howsmon et al. (17) on ovine anterior leaflets showing low-grade ischemic MR eight weeks post myocardial infarction. Due to leaflet tethering, the anterior leaflet fibers were permanently stretched in the radial direction resulting in a higher radial stiffness at low stress and a decrease in radial extensibility compared to the healthy ones. Long-term effects were not investigated in this study. In our study, we mainly observed a decrease in stiffness in the circumferential direction of MR anterior leaflets and a slight increase in radial stiffness.

### 4.3. Chord Morphology

#### 4.3.1. Basal vs. Marginal, Anterior vs. Posterior

A large variability in chord diameter was found between the different valves. Figure 4A shows the diameter differences between the chordal groups for HC and MR valves. HC AB chords were thicker than AM and PB chords, whereas AM and PB chords were thicker than PM chords. However, these differences were not statistically significant. Zuo et al. (14) found that AM and PM chords were significantly smaller than AB and PB chords, respectively, but also no significant differences were observed between AB and PB chords and between AM and PM chords. Note that anterior strut chords were classified as a separate group in most studies, whereas these chords were included in the AB group in this paper. Also for the MR valves similar diameter differences were found between the different chord types. AB chords were thicker than AM and PB chords, whereas AM and PB chords were thicker than PM chords.

Larger diameter differences were found when grouping the chords per insertion region, i.e., basal and marginal, and per leaflet type, i.e., anterior and posterior. Anterior chords were thicker than posterior chords (*p* < 0.01 for MR) and basal chords were thicker than marginal chords (*p* < 0.05 for HC) for both HC and MR valves as shown in Figures 4B,C, respectively. Liao et al. (24) found the same relation between the basal and marginal chords in their study, but no distinction was made between the anterior and posterior chords. Besides the difference in classification of the anterior chords, slightly larger diameters were found by Zuo et al., namely 0.71±0.18mm for the posterior chords compared to our 0.43±0.15mm. However, this can be related to the different methods (optical microscope vs digital camera) that were used in the different studies.

#### 4.3.2. Healthy vs. Diseased

Larger chord diameters were observed in the MR group compared to the HC group as seen in Figure 4, but these differences were not statistically significant due to the large diameter variation within each category. Also when grouping the chords per insertion region and per leaflet type, an increase in chord diameter was found for all MR chord groups and this difference was significant for the marginal chords (*p* < 0.05).

Grande-Allen et al. (15) found the opposite trend in their study: chords of hearts with dilated and ischemic CMP had a slightly smaller cross-sectional area than HC chords. No distinction was however made between chordal types in their study and as supported by Figure 4A, clear diameter differences exist between the different chord types of both HC and MR valves. Hence, grouping all the chords together might cancel the difference between MR and HC chords. Also a different measuring technique was used in their study. The cross-sectional area was calculated from the assumed density of the chords and their measured length and weight after testing. To the best of the author's knowledge, this is the first study analyzing the differences between HC and MR chords, distinguishing between AB, AM, PB and PM chords.

### 4.4. Chord Mechanics

#### 4.4.1. Basal vs. Marginal, Anterior vs. Posterior

A third order Ogden model was used to describe the nonlinear mechanical behavior of the chord samples. Figure 5B shows the fitting for a representative chord sample. The resulting stress-stretch curves of the HC and MR groups are given in Figures 7A,B, respectively. The posterior chord group (PB and PM) showed a smaller stiffness variance at low stress compared to the anterior group (AB and AM) for both HC and MR valves.

Also, for the chord samples, stiffness at low and high stress was calculated. The boxplots in Figures 10A,B show a large variance in stiffness and hence only insignificant stiffness differences between chordal types were found. At high stress, PB chords were stiffer than PM and AB chords for both HC and MR valves. Further, AM chords were stiffer than AB and PM chords for the HC valves, whereas they were more compliant than PM chords for the MR valves. The same differences were observed at low stress, but to a lower extent.

Some general trends are seen when grouping the chords based on insertion region and leaflet type. Posterior chords were stiffer than anterior chords at high stress for both HC and MR valves and this difference was significant for the MR group (*p* < 0.05) as seen in Figure 10D. Figure 10C also shows the same trend for the MR group at low stress, but no difference was observed for the HC group. Further, marginal chords were slightly stiffer than basal chords at high stress, but no clear difference was observed at low stress as seen in Figures 10E,F.

These findings are in agreement with previous studies on human and porcine mitral valve chords: thinner marginal chords were stiffer and less extensible than thicker basal chords (11, 14, 24, 25). Liao et al. (24) attributed these chordal mechanical differences to a different collagen fibril crimp period and fibril configuration. Collagen fibrils in the thicker basal chords were more highly crimped than in the thinner marginal chords, increasing their extensibility. On the other hand, larger fibril diameters and a smaller average fibril density were found in basal chords compared to marginal chords, resulting in a lower amount of interfibrillar linkages and lower stiffness. Zuo et al. (14) also examined the difference in mechanical properties between human anterior and posterior chords. Similar as our results, posterior chords were stiffer than anterior chords at high load. One has to note that a different chordal classification was used compared to this article. We also included anterior strut chords in the anterior group, which were found to be the most compliant chords, whereas Zuo et al. only included basal and marginal chords in that group. Pokutta-Paskaleva et al. (11) did not find significant differences in mechanical properties between porcine anterior and posterior chords.

#### 4.4.2. Healthy vs. Diseased

The boxplots in Figures 10A,B do not show significant stiffness differences between the MR and HC chord samples at both low and high stress. After all, there is a large variance in observed stiffness and the sample size is limited for some chordal groups due to exclusion of some samples. AM MR chords were less stiff than HC chords at low stress, whereas AB and PB chords were slightly stiffer in MR than HC. Larger differences were seen at high stress: MR AB and AM chords were more compliant than the HC groups, whereas MR PM chords were slightly stiffer than HC PM chords. No difference was observed between MR and HC PB chords.

A trend becomes more clear grouping the chords per insertion region or leaflet type. Diseased basal and marginal chords were less stiff than healthy ones at both low and high stress as shown in Figures 10E,F. Figures 10C,D show the same trend for the anterior chords at low stress and for the anterior and posterior chords at high stress; the MR chords were here also more compliant than the HC chords. The opposite was found for the posterior chords at low stress.

Similar as in this study, Prot et al. (16) reported more compliant chords in CMP compared to HC valves. However, this difference was only based on two valves. An opposite trend was observed by Grande-Allen et al. (15). Uniaxial experiments showed slightly stiffer chords in MR than HC. However, as already mentioned before, no difference was made between anterior and posterior or basal and marginal chords in this study.

### 4.5. Clinical Relevance

Previous studies already suggested that tethering of the mitral valve leaflets due to ventricular remodeling induces leaflet growth and thickening (4, 6). Our results show now that the left ventricular remodeling seen in CMP also induces critical changes in mitral valve leaflet mechanical behavior. The chords on the other hand, although thicker and less stiff in MR, are less affected by papillary muscle displacement and annular dilation.

These findings are of great importance for the understanding and treatment of secondary MR. Finite element models of mitral valves with secondary MR are currently based on healthy biaxial porcine or human leaflet data and healthy human or porcine chord data (26, 27). Our results did not reveal significant stiffness differences between MR and HC chords, but the change in leaflet tissue anisotropy observed in MR should be incorporated in computational models of secondary MR. Further, this change in leaflet material properties should also be taken into account in treatment strategies. The circumferential direction of the leaflets is more compliant in patients with secondary MR, reducing its load bearing capacity, whereas the radial direction of the leaflets is slightly stiffer in secondary MR, making it harder to extent the leaflet in this direction. These differences in mechanical behavior should be taken into account in MV surgery.

### 4.6. Study Limitations and Future Work

Some remarks should be made with respect to the mechanical experiments and data analysis.

#### Mechanical Experiments

No distinction was made between the different layers of the mitral valve leaflets and leaflets were assumed to be homogeneous through the thickness. Further, only the central region of the valve leaflets was mechanically tested and these properties might not be representative for the entire valve leaflet. Full field strain and thickness measurements were obtained for the entire sample, but only the average values were used in the data processing.

The strain measurement of the chord samples was based on the displacement of the clamps as marker tracking and DIC measurements were not feasible due to the small sample area. This method does not take into account slipping of the sample out of the clamps, which might cause an overestimation of the sample's extensibility and an underestimation of its stiffness. Therefore, when slip was noticed during experiments, data from a lower strain level for which no slip occurred were used or the sample was excluded from the analysis.

#### Parameter Fitting

The applied preload before the stretch cycles was relatively large compared to the maximum load and could not be ignored. Therefore, prestretch parameters were introduced as optimization variables and the parameter fitting was performed for a range of possible prestretch values. The parameter combination with the lowest NRMSE was considered as the correct prestretch. The MN and Ogden parameters resulting from the optimization problem sometimes reached their limit value as can be seen in Supplementary Tables 6–11. This prestretch fitting requires further research, but this was not the goal of this paper. For this, we refer to Vander Linden et al. (21).

The biaxial specimens were tested along five ratios, however only data of ratio (*circ:rad*) 1:1 were used to determine the MN material parameters as the preload correction was not able to fit five ratios simultaneously. As ratio 1:1 might not correspond to the physiological situation, future work includes parameter fitting based on the five ratios to capture the full biaxial behavior.

The Ogden model was able to capture the mechanical behavior of the chord samples very well within the range of the experimental values. Extrapolation of the fitting to values from one to prestretch-value sometimes resulted in nonphysical solutions, such as negative stresses for stretches larger than one. Therefore, the most optimal solution with a physiological response between stretch one and the maximal stretch was chosen as the correct solution for the chord samples.

#### Data Analysis

Linearized stiffness was defined at both low and high stress to quantify the nonlinear mechanical behavior. The last data points of the stress-stretch curves were considered as the post-transitional region in the stress-stretch curves. This can result in measurement variations between the samples as different strain levels were reached for each experiment. Several options were considered to quantify the mechanical response and calculating the slopes based on the first and last data points was the most consistent method.

The statistical analysis was sometimes based on a small sample size due to sample exclusions and results should be treated carefully. More samples should be included to take into account the patient variability and to be able to make a thorough statistical analysis. Also, no correlation was made between the results and the corresponding donor characteristics. No echocardiographic data of the donors were obtained and hence no detailed information about mitral valve function and the severity of secondary mitral regurgitation was available.

#### Microstructural Analysis

Finally, in this work, it was not possible to perform histological analysis of the samples as well, besides the mechanical analysis. Combined histological analysis and mechanical experiments would give a more profound understanding of the microstructural adaptations supporting the change in tissue anisotropy in MR. Future work includes also histological analysis of human healthy mitral valves and mitral valves with secondary MR.

## 5. Conclusion

Secondary MR originates from a left ventricular disease, in which the altered ventricular geometry affects the subvalvular apparatus supporting the mitral valve. Also active valvular remodeling is seen as response to the altered loading pattern, influencing the valve mechanical properties.

In this work, we investigated these pathophysiological changes in the mechanical behavior of mitral valve leaflets and chords in secondary MR as opposed to healthy control. Planar biaxial tensile tests were performed on healthy and diseased mitral valve leaflets and uniaxial tensile tests on different categories of mitral valve chords.

Posterior leaflet samples were significantly thicker and more compliant in both circumferential and radial directions at low and high stress than anterior leaflet samples. Anterior leaflets had an AI larger than one, indicating a stiffer circumferential than radial direction, whereas the AI of posterior leaflets was slightly smaller than one, resulting in more or less isotropic samples. Further, basal and anterior chords had a larger diameter and were more compliant at high stress than marginal and posterior chords, respectively.

Also pathophysiological changes were seen after left ventricular remodeling. Both anterior and posterior leaflets were slightly thicker in MR compared to the HC samples. A clear difference between MR and HC anterior and posterior leaflet samples was found based on the AI. A more compliant circumferential and stiffer radial direction resulted in an AI smaller than one for both leaflets. Hence, MR leaflet samples were highly anisotropic with the radial direction being stiffest. Grouped per leaflet type and insertion region, MR chords were thicker and less stiff at high stress than HC chords.

These findings show that the mitral valve adapts to the changes in loading pattern due to left ventricular remodeling. This change in mechanical behavior gives more insight into the mechanisms behind secondary MR and should be taken into account when evaluating treatment strategies.

## Data Availability Statement

The dataset presented in this study is publicly available in KU Leuven RDR. The data can be found here: https://rdr.kuleuven.be/dataset.xhtml?persistentId=doi:10.48804/Q3LPZ9.

## Ethics Statement

The studies involving human participants were reviewed and approved by Comité d'Ethique Hospitalo-Facultaire Saint-Luc-UCL (CEHF). The patients/participants provided their written informed consent to participate in this study.

## Author Contributions

HF, NF, PaV, PiV, FR, PB, and SD contributed to conception and design of the study. RJ collected the human mitral valves. PaV, KV, and HF performed the testing and data analysis. PaV wrote the manuscript. SD, PB, FR, PiV, and RJ provided clinical expertise. SD, PaV, HF, NF, and JV collected funding. HF, NF, and JV supervised the project. All authors contributed to the article and approved the submitted version.

## Funding

This work was supported by Fonds voor Hartchirurgie, by KU Leuven through a category 2 research project (C2-ADAPT) and by Research Foundation Flanders (FWO) through a predoctoral fellowship fundamental research to PaV (11H5821N), a predoctoral fellowship strategic basic research to KV (SB1SA9119N), and a junior postdoctoral fellowship to HF (12ZC820N).

## Conflict of Interest

The authors declare that the research was conducted in the absence of any commercial or financial relationships that could be construed as a potential conflict of interest.

## Publisher's Note

All claims expressed in this article are solely those of the authors and do not necessarily represent those of their affiliated organizations, or those of the publisher, the editors and the reviewers. Any product that may be evaluated in this article, or claim that may be made by its manufacturer, is not guaranteed or endorsed by the publisher.

## Abbreviations

A: anterior
AB: anterior basal
AI: anisotropy index
AL: anterior leaflet
AM: anterior marginal
B: basal
Circ: circumferential
CMP: cardiomyopathy
DIC: digital image correlation
DMSO: dimethyl sulfoxide
HC: healthy control
HS: high stress
LS: low stress
M: marginal
MN: May-Newman and Yin
MR: mitral regurgitation
MV: mitral valve
NRMSE: normalized root mean square error
P: posterior
PB: posterior basal
PL: posterior leaflet
PM: posterior marginal
Rad: radial.

## References

[1] Levine RA Hagége AA Judge DP Padala M Dal-Bianco JP Aikawa E. Mitral valve disease-morphology and mechanisms. Nat Rev Cardiol. (2015) 12:689–710. 10.1038/nrcardio.2015.161 26483167 PMC4804623

[2] Nkomo VT Gardin JM Skelton TN Gottdiener JS Scott CG Enriquez-Sarano M. Burden of valvular heart diseases: a population-based study. Lancet. (2006) 368:1005–11. 10.1016/S0140-6736(06)69208-8 16980116

[3] Bertrand PB Schwammenthal E Levine RA Vandervoort PM. Exercise dynamics in secondary mitral regurgitation: pathophysiology and therapeutic implications. Circulation. (2017) 135:297–314. 10.1161/CIRCULATIONAHA.116.025260 28093494 PMC5245732

[4] Rausch MK Tibayan FA Craig Miller D Kuhl E. Evidence of adaptive mitral leaflet growth. J Mech Behav Biomed Mater. (2012) 15:208–17. 10.1016/j.jmbbm.2012.07.001 23159489 PMC3508091

[5] Dal-Bianco JP Aikawa E Bischoff J Guerrero JL Handschumacher MD Sullivan S. Active adaptation of the tethered mitral valve: insights into a compensatory mechanism for functional mitral regurgitation. Circulation. (2009) 120:334–42. 10.1161/CIRCULATIONAHA.108.846782 19597052 PMC2752046

[6] Grande-Allen KJ Borowski AG Troughton RW Houghtaling PL Dipaola NR Moravec CS. Apparently normal mitral valves in patients with heart failure demonstrate biochemical and structural derangements: An extracellular matrix and echocardiographic study. J Am Coll Cardiol. (2005) 45:54–61. 10.1016/j.jacc.2004.06.079 15629373

[7] May-Newman K Yin F. Biaxial mechanical behavior of excised porcine mitral valve leaflets. Am J Physiol Heart Circul Physiol. (1995) 269:H1319–27. 10.1152/ajpheart.1995.269.4.H1319 7485564

[8] Laurence D Ross C Jett S Johns C Echols A Baumwart R. An investigation of regional variations in the biaxial mechanical properties and stress relaxation behaviors of porcine atrioventricular heart valve leaflets. J Biomech. (2019) 83:16–27. 10.1016/j.jbiomech.2018.11.015 30497683 PMC8008702

[9] Jett S Laurence D Kunkel R Babu AR Kramer K Baumwart R. An investigation of the anisotropic mechanical properties and anatomical structure of porcine atrioventricular heart valves. Journal of the Mech Behav Biomed Mater. (2018) 87:155–71. 10.1016/j.jmbbm.2018.07.024 30071486 PMC8008704

[10] Grashow JS Yoganathan AP Sacks MS. Biaixal stress-stretch behavior of the mitral valve anterior leaflet at physiologic strain rates. Ann Biomed Eng. (2006) 34:315–25. 10.1007/s10439-005-9027-y 16450193

[11] Pokutta-Paskaleva A Sulejmani F DelRocini M Sun W. Comparative mechanical, morphological, and microstructural characterization of porcine mitral and tricuspid leaflets and chordae tendineae. Acta Biomater. (2019) 85:241–52. 10.1016/j.actbio.2018.12.029 30579963 PMC6344049

[12] Pham T Sun W. Material properties of aged human mitral valve leaflets. J Biomed Mater Res Part A. (2014) 102:2692–703. 10.1002/jbm.a.34939 24039052 PMC4033712

[13] Pham T Sulejmani F Shin E Wang D Sun W. Quantification and comparison of the mechanical properties of four human cardiac valves. Acta Biomater. (2017) 54:345–55. 10.1016/j.actbio.2017.03.026 28336153

[14] Zuo K Pham T Li K Martin C He Z Sun W. Characterization of biomechanical properties of aged human and ovine mitral valve chordae tendineae. J Mech Behav Biomed Mater. (2016) 62:607–18. 10.1016/j.jmbbm.2016.05.034 27315372 PMC5058331

[15] Grande-Allen KJ Barber JE Klatka KM Houghtaling PL Vesely I Moravec CS. Mitral valve stiffening in end-stage heart failure: evidence of an organic contribution to functional mitral regurgitation. J Thorac Cardiovasc Surg. (2005) 130:783–90. 10.1016/j.jtcvs.2005.04.019 16153929

[16] Prot V Skallerud B Sommer G Holzapfel GA. On modelling and analysis of healthy and pathological human mitral valves: two case studies. J Mech Behav Biomed Mater. (2010) 3:167–77. 10.1016/j.jmbbm.2009.05.004 20129416

[17] Howsmon DP Rego BV Castillero E Ayoub S Khalighi AH Gorman RC. Mitral valve leaflet response to ischaemic mitral regurgitation: from gene expression to tissue remodelling. J R Soc Interface. (2020) 17:20200098. 10.1098/rsif.2020.0098 32370692 PMC7276554

[18] May-Newman K Yin FCP. A constitutive law for mitral valve tissue. J Biomech Eng. (1998) 130:38–47. 10.1115/1.2834305 9675679

[19] Ogden RW. Non-linear Elastic Deformations. New York, NY: Dover Publications (1997).

[20] Ogden RW Saccomandi G Sgura I. Fitting hyperelastic models to experimental data. Comput Mech. (2004) 34:484–502. 10.1007/s00466-004-0593-y

[21] Vander Linden K Fehervary H Maes L Famaey N. An improved parameter fitting approach of a planar biaxial test including the experimental preload. In preparation. 10.1016/j.jmbbm.2022.105389 35932647

[22] Andersson JAE Gillis J Horn G Rawlings JB Diehl M. CasADi-A software framework for nonlinear optimization and optimal control. Math Prog Comput. (2019) 11:1–36. 10.1007/s12532-018-0139-4

[23] Roberts N Morticelli L Jin Z Ingham E Korossis S. Regional biomechanical and histological characterization of the mitral valve apparatus: implications for mitral repair strategies. J Biomech. (2016) 49:2491–501. 10.1016/j.jbiomech.2015.12.042 26787008

[24] Liao J Vesely I. A structural basis for the size-related mechanical properties of mitral valve chordae tendineae. J Biomech. (2003) 36:1125–33. 10.1016/S0021-9290(03)00109-X 12831738

[25] Kunzelman KS Cochran RP. Mechanical properties of basal and marginal mitral valve chordae tendineae. ASAIO Trans. (1990) 36:M405–8. 2252712

[26] Pham T Kong F Martin C Wang Q Primiano C Mckay R. Finite element analysis of patient-specific mitral valve with mitral regurgitation. Cardiovasc Eng Technol. (2017) 8:3–16. 10.1007/s13239-016-0291-9 28070866 PMC5321865

[27] Wenk JF Zhang Z Cheng G Malhotra D Acevedo-Bolton G Burger M. First finite element model of the left ventricle with mitral valve: insights into ischemic mitral regurgitation. Ann Thorac Surg. (2010) 89:1546–54. 10.1016/j.athoracsur.2010.02.036 20417775 PMC2887313

