# Supplementary material for: Alterations in Human Mitral Valve Mechanical Properties Secondary to Left Ventricular Remodeling: A Biaxial Mechanical Study
Source: Front Cardiovasc Med. 2022 Jun 9;9:876006. doi: 10.3389/fcvm.2022.876006 (PMC9258718; doi:10.3389/fcvm.2022.876006)
Supplement: Supplementary file 1 [file Data_Sheet_1.pdf]

frontiers

Supplementary Material

1 LEAFLET MORPHOLOGY

Table S1. Thickness of the leaflet samples for each group. Data are represented as mean ± standard deviation. The amount of samples is indicated between brackets.

	**AL** [mm]	**PL** [mm]
**HC**	0.94 ± 0.17 (10)	1.83 ± 0.34 (10)
**MR**	1.10 ± 0.46 (8)	2.01 ± 0.61 (8)

2 LEAFLET MECHANICS

Table S2. Stiffness of the leaflet samples at low (LS) and high stress (HS). Data are represented as median ± interquartile range. The amount of samples is indicated between brackets.

		**AL**		**PL**	
		**Circ**	**Rad**	**Circ**	**Rad**
**Low stress** [kPa]	**HC**	60.66 ± 40.58 (9)	37.56 ± 26.83 (9)	4.41 ± 2.89 (9)	4.62 ± 4.21 (9)
	**MR**	14.93 ± 26.31 (8)	53.56 ± 42.33 (8)	3.85 ± 6.36 (5)	6.40 ± 24.27 (5)
**High stress** [MPa]	**HC**	9.39 ± 11.79 (9)	7.40 ± 4.23 (9)	1.31 ± 0.64 (9)	1.31 ± 1.41 (9)
	**MR**	3.82 ± 6.57 (8)	10.52 ± 4.58 (8)	0.62 ± 1.04 (5)	1.90 ± 3.50 (5)

Table S3. Anisotropy index of the leaflet samples at low (LS) and high stress (HS). Data are represented as median ± interquartile range. The amount of samples is indicated between brackets.

		**AL**	**PL**
**Low stress** [-]	**HC**	1.14 ± 0.81 (9)	0.91 ± 0.83 (9)
	**MR**	0.62 ± 0.58 (8)	0.41 ± 0.42 (5)
**High stress** [-]	**HC**	1.19 ± 1.02 (9)	0.89 ± 1.05 (9)
	**MR**	0.61 ± 0.54 (8)	0.40 ± 0.42 (5)

3 CHORD MORPHOLOGY

Table S4. Diameter of the chord samples grouped per chordal type, leaflet type and insertion region. Data are represented as mean ± standard deviation. The amount of samples is indicated between brackets.

		**Basal** [mm]	**Marginal** [mm]	
**Anterior**	**HC**	0.75 ± 0.39 (10)	0.46 ± 0.21 (10)	0.61 ± 0.34 (20)
	**MR**	1.04 ± 0.19 (8)	0.73 ± 0.30 (8)	0.88 ± 0.29 (16)
**Posterior**	**HC**	0.50 ± 0.15 (10)	0.36 ± 0.12 (10)	0.43 ± 0.15 (20)
	**MR**	0.64 ± 0.32 (8)	0.48 ± 0.09 (8)	0.56 ± 0.24 (16)
	**HC**	0.63 ± 0.32 (20)	0.41 ± 0.17 (20)	
	**MR**	0.84 ± 0.33 (16)	0.60 ± 0.25 (16)	

4 CHORD MECHANICS

Table S5. Stiffness of the chord samples at low (LS) and high stress (HS) grouped per chordal type, leaflet type and insertion region. Data are represented as median ± interquartile range. The amount of samples is indicated between brackets.

		**Low stress** [MPa]		
		**Basal**	**Marginal**	
**Anterior**	**HC**	11.23 ± 33.69 (7)	56.01 ± 55.04 (8)	38.23 ± 56.02 (15)
	**MR**	17.70 ± 22.46 (6)	19.89 ± 34.55 (8)	19.89 ± 19.93 (14)
**Posterior**	**HC**	49.66 ± 40.33 (8)	37.99 ± 50.78 (7)	38.20 ± 48.12 (15)
	**MR**	58.83 ± 25.65 (4)	37.56 ± 34.00 (6)	51.24 ± 35.99 (10)
	**HC**	38.23 ± 37.15 (15)	38.20 ± 47.77 (15)	
	**MR**	26.31 ± 46.53 (10)	22.52 ± 39.68 (14)	

		**High stress** [MPa]		
		**Basal**	**Marginal**	
**Anterior**	**HC**	143.10 ± 177.57 (7)	295.53 ± 255.74 (8)	150.40 ± 266.23 (15)
	**MR**	72.29 ± 49.30 (6)	103.39 ± 50.80 (8)	100.73 ± 61.44 (14)
**Posterior**	**HC**	263.76 ± 130.51 (8)	116.45 ± 191.92 (7)	224.69 ± 179.48 (15)
	**MR**	264.06 ± 130.40 (4)	138.25 ± 142.53 (6)	204.52 ± 142.53 (10)
	**HC**	217.54 ± 192.67 (15)	224.69 ± 217.73 (15)	
	**MR**	106.99 ± 203.58 (10)	118.73 ± 44.43 (14)	

5 OVERVIEW LEAFLET SAMPLES

The processed first Piola-Kirchhoff stress-stretch data of every sample can be found here: https://rdr.kuleuven.be/dataset.xhtml?persistentId=doi:10.48804/Q3LPZ9.

Table S6. Overview of the anterior leaflet results. The thickness (t), stiffness at low and high stress (S^{LS}, S^{HS}), anisotropy index at low and high stress (AI^{LS}, AI^{HS}) and fitting parameters (c_{10}, c_1, c_2, α, G_{11}, G_{22}, NRMSE) are given for each sample. Parameters indicated in bold are reaching their limit value.

Sample	t [mm]	S^{LS}_{circ} [kPa]	S^{LS}_{rad} [kPa]	AI^{LS} [-]	S^{HS}_{circ} [MPa]	S^{HS}_{rad} [MPa]	AI^{HS} [-]	c_{10} [kPa]	c_1 [-]	c_2 [-]	α [rad]	G_{11} [-]	G_{22} [-]	NRMSE [-]
**HC 1**	1.00 ± 0.15	-	-	-	-	-	-	-	-	-	-	-	-	-
**HC 2**	0.99 ± 0.30	62.11	54.59	1.14	9.39	7.92	1.19	132.50	12.50	359.48	-0.59	1.00	1.07	0.62
**HC 3**	1.25 ± 0.34	19.76	24.93	0.79	4.71	6.17	0.76	22.80	8.96	1745.70	-0.88	1.01	1.07	0.45
**HC 4**	0.87 ± 0.28	25.71	37.56	0.68	4.18	6.23	0.67	998.20	0.12	96.92	0.90	1.09	1.00	0.84
**HC 5**	1.00 ± 0.23	21.01	22.08	0.95	6.10	6.59	0.92	13.00	23.19	80.17	-1.55	0.97	1.09	0.60
**HC 6**	1.13 ± 0.23	31.26	34.20	0.91	6.60	7.40	0.89	25.00	28.09	1623.16	-0.86	1.10	1.01	0.81
**HC 7**	0.79 ± 0.23	61.68	49.86	1.24	17.24	13.34	1.29	21.70	70.84	**5000.00**	0.63	1.04	1.03	0.56
**HC 8**	0.72 ± 0.25	60.66	14.62	4.15	14.69	2.59	5.68	43.70	7.63	1674.87	0.06	1.02	1.10	1.61
**HC 9**	0.88 ± 0.33	74.14	44.73	1.66	18.43	9.48	1.94	43.60	23.93	1297.49	0.25	1.04	1.05	0.50
**HC 10**	0.71 ± 0.18	136.38	76.10	1.79	26.93	14.49	1.86	188.60	4.05	2322.03	0.61	0.99	1.10	0.97
**MR 1**	1.16 ± 0.13	24.89	30.06	0.83	8.18	10.21	0.80	18.70	11.95	2226.90	0.87	1.09	0.99	0.59
**MR 2**	1.27 ± 0.38	11.63	45.87	0.25	1.99	7.94	0.25	143.90	0.91	3478.34	1.12	1.10	0.99	1.35
**MR 3**	0.66 ± 0.18	44.53	66.86	0.67	7.81	11.76	0.66	265.60	0.48	2036.54	-0.89	1.08	0.98	1.07
**MR 4**	0.89 ± 0.28	43.04	74.03	0.58	7.75	13.65	0.57	261.20	0.71	706.08	0.94	1.09	0.99	1.34
**MR 6**	0.92 ± 0.23	18.23	19.32	0.94	5.65	6.01	0.94	16.20	4.17	2744.94	-0.80	0.97	1.10	0.53
**MR 7**	0.88 ± 0.09	6.49	67.17	0.10	0.97	11.36	0.08	**1000.00**	0.28	330.63	-1.37	1.06	1.02	1.06
**MR 9**	0.90 ± 0.35	8.82	61.24	0.14	1.46	10.84	0.13	234.70	0.40	749.46	1.25	1.07	1.02	1.40
**MR 10**	2.14 ± 0.45	5.88	8.04	0.73	0.67	1.04	0.65	**1000.00**	0.11	4.51	-1.15	1.04	1.06	1.49

Table S7. Overview of the posterior leaflet results. The thickness (t), stiffness at low and high stress (S^{LS}, S^{HS}), anisotropy index at low and high stress (AI^{LS}, AI^{HS}) and fitting parameters (c_{10}, c_1, c_2, α, G_{11}, G_{22}, NRMSE) are given for each sample. Parameters indicated in bold are reaching their limit value.

**Sample**	t [mm]	S_{circ}^{LS} [kPa]	S_{rad}^{LS} [kPa]	AI^{LS} [-]	S_{circ}^{HS} [MPa]	S_{rad}^{HS} [MPa]	AI^{HS} [-]	c_{10} [kPa]	c_1 [-]	c_2 [-]	α [rad]	G_{11} [-]	G_{22} [-]	**NRMSE** [-]
**HC 1**	1.30 ± 0.36	4.41	4.99	0.88	1.58	1.88	0.84	2.10	35.17	1023.06	0.97	1.08	1.00	1.07
**HC 2**	1.66 ± 0.56	5.84	8.20	0.71	0.95	1.35	0.70	647.70	0.47	201.42	0.90	1.00	1.03	1.55
**HC 3**	2.31 ± 0.94	5.37	5.65	0.95	1.23	1.31	0.94	3.00	50.59	2157.44	−0.84	1.00	1.07	0.59
**HC 4**	2.06 ± 0.85	2.16	2.37	0.91	0.33	0.37	0.89	5.20	14.61	913.25	0.86	1.03	1.04	0.44
**HC 5**	2.10 ± 0.89	3.66	4.62	0.79	1.35	1.81	0.75	0.80	222.91	**5000.00**	−1.28	1.04	1.03	1.16
**HC 6**	1.69 ± 0.29	-	-	-	-	-	-	-	-	-	-	-	-	-
**HC 7**	1.36 ± 0.56	10.28	9.34	1.10	1.94	1.75	1.11	21.10	3.96	1407.14	0.75	0.99	1.09	0.78
**HC 8**	2.02 ± 0.75	3.35	0.97	3.47	1.31	0.32	4.15	1.10	15.15	4522.73	0.32	1.03	1.10	1.30
**HC 9**	2.14 ± 0.78	7.11	1.22	5.84	1.55	0.20	7.87	6.30	4.92	1797.72	−0.11	1.00	1.10	2.57
**HC 10**	1.65 ± 0.59	3.00	3.34	0.90	0.83	0.95	0.87	3.20	7.64	888.79	0.84	1.02	1.10	1.54
**MR 1**	2.55 ± 0.63	-	-	-	-	-	-	-	-	-	-	-	-	-
**MR 2**	1.36 ± 0.37	3.85	25.93	0.15	0.52	4.29	0.12	997.40	0.14	73.37	1.39	1.07	1.03	2.35
**MR 3**	1.32 ± 0.24	4.24	4.51	0.94	0.93	1.00	0.93	6.60	4.94	988.22	−0.81	1.09	1.02	0.37
**MR 4**	2.63 ± 0.78	-	-	-	-	-	-	-	-	-	-	-	-	-
**MR 6**	2.28 ± 0.56	-	-	-	-	-	-	-	-	-	-	-	-	-
**MR 7**	2.76 ± 0.52	1.44	6.40	0.23	0.37	1.90	0.20	3.80	3.74	2438.46	−1.22	1.07	1.04	0.56
**MR 9**	1.51 ± 0.44	19.50	37.15	0.52	3.29	6.79	0.48	50.20	9.88	2120.84	−1.05	1.01	1.04	0.46
**MR 10**	1.66 ± 0.47	1.78	4.35	0.41	0.62	1.55	0.40	6.80	0.14	428.15	1.01	1.09	1.09	0.51

6 OVERVIEW CHORD SAMPLES

The processed first Piola-Kirchhoff stress-stretch data of every sample can be found here: https://rdr.kuleuven.be/dataset.xhtml?persistentId=doi:10.48804/Q3LPZ9.

Table S8. Overview of the anterior basal chord results. The diameter (d), stiffness at low and high stress (S^{LS}, S^{HS}) and fitting parameters (μ_1, a_1, μ_2, a_2, μ_3, a_3, G_{22}, NRMSE) are given for each sample. Parameters indicated in bold are reaching their limit value.

Sample	d [mm]	S^{LS} [MPa]	S^{HS} [MPa]	μ_1 [MPa]	a_1 [-]	μ_2 [MPa]	a_2 [-]	μ_3 [MPa]	a_3 [-]	G_{22} [-]	NRMSE [-]
**HC 1**	0.93 ± 0.24	4.19	46.44	−13.24	−0.0002	−19.67	−0.0001	33.45	8.14	1.017	0.27
**HC 2**	0.46 ± 0.12	39.10	249.28	−40.40	−138.51	24.23	48.22	16.59	68.18	1.013	0.23
**HC 3**	1.38 ± 0.23	11.23	48.63	10.71	27.71	−7.97	−33.96	−0.03	**−250.00**	1.007	0.16
**HC 4**	0.94 ± 0.28	-	-	-	-	-	-	-	-	-	-
**HC 5**	1.28 ± 0.54	4.15	31.05	3.71	44.07	−1.60	−104.29	−1.86	−102.16	1.010	0.22
**HC 6**	0.42 ± 0.15	98.19	443.74	22.81	80.44	−37.12	−174.07	33.27	50.43	1.004	0.28
**HC 7**	0.43 ± 0.25	38.23	150.40	14.29	24.90	−17.89	−157.60	8.93	78.78	1.018	0.17
**HC 8**	0.21 ± 0.10	-	-	-	-	-	-	-	-	-	-
**HC 9**	0.57 ± 0.21	-	-	-	-	-	-	-	-	-	-
**HC 10**	0.91 ± 0.43	8.20	143.10	3.64	20.77	−1.12	−49.22	−1.13	−49.22	1.050	0.45
**MR 1**	1.40 ± 0.33	-	-	-	-	-	-	-	-	-	-
**MR 2**	0.73 ± 0.28	182.24	293.88	0.01	125.61	58.74	14.03	−0.03	**−249.98**	1.011	0.33
**MR 3**	0.95 ± 0.35	-	-	-	-	-	-	-	-	-	-
**MR 4**	1.12 ± 0.23	5.78	52.99	−0.59	−236.16	0.27	118.96	1.67	53.88	1.018	0.34
**MR 6**	1.15 ± 0.36	8.37	53.32	−6.88	−185.13	3.35	40.30	3.40	92.57	1.010	0.20
**MR 7**	1.01 ± 0.35	30.83	83.24	−3.41	−212.94	0.39	123.47	11.17	59.09	1.006	0.23
**MR 9**	0.95 ± 0.21	21.80	102.62	−12.92	−212.80	4.13	111.29	10.64	67.23	1.011	0.25
**MR 10**	1.02 ± 0.46	13.60	61.34	5.96	45.36	3.15	71.51	−7.72	−142.62	1.009	0.49

Table S9. Overview of the anterior marginal chord results. The diameter (d), stiffness at low and high stress (S^{LS}, S^{HS}) and fitting parameters (μ_1, a_1, μ_2, a_2, μ_3, a_3, G_{22}, NRMSE) are given for each sample.

Sample	d [mm]	S^{LS} [MPa]	S^{HS} [MPa]	μ_1 [MPa]	a_1 [-]	μ_2 [MPa]	a_2 [-]	μ_3 [MPa]	a_3 [-]	G_{22} [-]	NRMSE [-]
**HC 1**	0.67 ± 0.11	19.88	102.02	−7.91	−76.20	−7.91	−76.20	17.48	31.82	1.010	0.16
**HC 2**	0.40 ± 0.14	52.03	312.85	−18.78	−98.98	42.63	41.43	−18.78	−98.98	1.012	0.20
**HC 3**	0.48 ± 0.15	-	-	-	-	-	-	-	-	-	-
**HC 4**	0.93 ± 0.44	6.13	30.13	−4.66	−208.77	2.33	104.39	2.13	27.48	1.012	0.60
**HC 5**	0.44 ± 0.09	63.55	278.21	25.05	33.91	13.79	67.52	−31.79	−133.14	1.011	0.25
**HC 6**	0.32 ± 0.11	76.69	457.42	−48.84	−186.61	30.40	46.02	22.37	94.12	1.008	0.33
**HC 7**	0.30 ± 0.10	72.53	352.09	61.08	21.70	−27.11	−51.92	−27.11	−51.92	1.016	0.31
**HC 8**	0.30 ± 0.06	-	-	-	-	-	-	-	-	-	-
**HC 9**	0.26 ± 0.04	60.00	318.27	−36.37	−59.78	72.47	25.35	−36.34	−59.80	1.025	0.25
**HC 10**	0.56 ± 0.18	5.64	56.87	−0.90	−55.83	−0.90	−55.83	2.97	24.10	1.005	0.50
**MR 1**	0.29 ± 0.09	116.23	278.20	46.24	45.20	−6.74	−151.15	−6.74	−151.15	1.003	0.16
**MR 2**	0.53 ± 0.13	77.57	114.76	29.47	13.48	20.88	99.27	−41.79	−198.53	1.016	0.19
**MR 3**	1.23 ± 0.37	10.90	51.73	5.76	53.61	4.52	89.29	−10.15	−179.01	1.008	0.18
**MR 4**	0.83 ± 0.32	20.75	100.06	11.14	47.72	−16.81	−154.87	6.09	80.00	1.005	0.18
**MR 6**	0.58 ± 0.17	18.46	138.64	−9.38	−80.37	−9.38	−80.38	18.50	34.99	1.020	0.18
**MR 7**	0.70 ± 0.12	19.02	101.39	−7.92	−75.72	−7.92	−75.72	17.33	31.70	1.008	0.14
**MR 9**	0.64 ± 0.29	20.90	105.38	−5.91	−80.20	−5.91	−80.19	14.80	32.78	1.009	0.21
**MR 10**	1.03 ± 0.25	5.78	22.58	−0.11	−99.15	1.49	4.09	0.45	35.56	1.033	0.16

Table S10. Overview of the posterior basal chord results. The diameter (d), stiffness at low and high stress (S^{LS}, S^{HS}) and fitting parameters (μ_1, a_1, μ_2, a_2, μ_3, a_3, G_{22}, NRMSE) are given for each sample.

**Sample**	$\boldsymbol{d}$ [mm]	$\boldsymbol{S^{LS}}$ [MPa]	$\boldsymbol{S^{HS}}$ [MPa]	$\boldsymbol{\mu_1}$ [MPa]	$\boldsymbol{a_1}$ [-]	$\boldsymbol{\mu_2}$ [MPa]	$\boldsymbol{a_2}$ [-]	$\boldsymbol{\mu_3}$ [MPa]	$\boldsymbol{a_3}$ [-]	$\boldsymbol{G_{22}}$ [-]	**NRMSE** [-]
**HC 1**	0.76 ± 0.18	23.54	87.55	7.21	95.77	8.53	40.37	−12.43	−198.16	1.005	0.36
**HC 2**	0.41 ± 0.06	115.18	269.96	14.01	55.21	42.57	30.93	−30.29	−115.03	1.012	0.18
**HC 3**	0.45 ± 0.11	51.86	286.04	−46.11	−168.64	22.58	39.68	22.19	84.55	1.009	0.32
**HC 4**	0.46 ± 0.14	-	-	-	-	-	-	-	-	-	-
**HC 5**	0.76 ± 0.20	26.55	83.99	−0.02	−183.06	8.36	28.57	−0.40	−66.35	1.009	0.39
**HC 6**	0.55 ± 0.18	32.94	217.54	15.88	46.16	13.51	76.68	−30.14	−152.71	1.007	0.27
**HC 7**	0.48 ± 0.18	88.29	280.08	−32.46	−126.69	48.89	48.01	0.08	107.00	1.004	0.33
**HC 8**	0.37 ± 0.13	-	-	-	-	-	-	-	-	-	-
**HC 9**	0.36 ± 0.07	49.08	257.56	66.93	19.75	−50.85	−9.90	−11.77	−71.18	1.004	0.21
**HC 10**	0.42 ± 0.21	50.23	314.57	13.21	70.43	−38.15	−135.98	25.84	44.30	1.000	0.41
**MR 1**	1.17 ± 0.27	-	-	-	-	-	-	-	-	-	-
**MR 2**	0.16 ± 0.07	-	-	-	-	-	-	-	-	-	-
**MR 3**	0.62 ± 0.23	65.55	264.91	6.80	41.27	6.80	41.27	6.80	41.27	1.002	1.30
**MR 4**	0.99 ± 0.42	-	-	-	-	-	-	-	-	-	-
**MR 6**	0.67 ± 0.24	16.85	111.36	16.00	44.78	−8.12	−102.78	−8.12	−102.78	1.002	0.26
**MR 7**	0.55 ± 0.25	57.53	263.21	61.80	46.71	−31.86	−113.08	−26.92	−113.08	1.014	0.20
**MR 9**	0.62 ± 0.18	-	-	-	-	-	-	-	-	-	-
**MR 10**	0.36 ± 0.09	60.13	370.45	57.70	35.08	−27.82	−82.21	−27.82	−82.21	1.008	0.26

Table S11. Overview of the posterior marginal chord results. The diameter (d), stiffness at low and high stress (S^{LS}, S^{HS}) and fitting parameters (μ_1, a_1, μ_2, a_2, μ_3, a_3, G_{22}, NRMSE) are given for each sample.

Sample	d [mm]	S^{LS} [MPa]	S^{HS} [MPa]	μ_1 [MPa]	a_1 [-]	μ_2 [MPa]	a_2 [-]	μ_3 [MPa]	a_3 [-]	G_{22} [-]	NRMSE [-]
**HC 1**	0.44 ± 0.15	30.44	116.45	30.92	20.79	-14.93	-49.57	-14.93	-49.57	1.020	0.09
**HC 2**	0.53 ± 0.12	37.99	106.61	13.97	112.84	16.00	7.26	-27.42	-226.22	1.017	0.62
**HC 3**	0.41 ± 0.16	-	-	-	-	-	-	-	-	-	-
**HC 4**	0.23 ± 0.06	96.65	321.09	-5.95	-78.28	-5.95	-78.28	38.37	25.68	1.014	0.12
**HC 5**	0.18 ± 0.04	110.00	489.99	-40.22	-58.92	-40.22	-58.92	89.97	24.63	1.008	0.44
**HC 6**	0.50 ± 0.14	15.61	100.37	12.11	55.45	-4.88	-133.56	-5.28	-133.41	1.011	0.22
**HC 7**	0.34 ± 0.09	38.20	224.69	-13.40	-78.41	-13.40	-78.41	30.46	32.92	1.015	0.25
**HC 8**	0.24 ± 0.06	-	-	-	-	-	-	-	-	-	-
**HC 9**	0.27 ± 0.06	-	-	-	-	-	-	-	-	-	-
**HC 10**	0.46 ± 0.10	33.75	104.55	11.47	22.73	10.04	114.50	-20.08	-228.99	1.010	0.32
**MR 1**	0.48 ± 0.12	-	-	-	-	-	-	-	-	-	-
**MR 2**	0.65 ± 0.17	117.95	122.71	44.54	0.0004	27.07	102.49	-54.07	-205.01	1.026	0.31
**MR 3**	0.56 ± 0.21	24.14	130.69	8.00	32.99	4.72	96.29	-9.55	-192.39	1.005	0.36
**MR 4**	0.45 ± 0.17	-	-	-	-	-	-	-	-	-	-
**MR 6**	0.44 ± 0.16	44.94	265.23	0.0048	81.61	43.11	39.92	-43.16	-92.53	1.007	0.26
**MR 7**	0.41 ± 0.16	30.19	108.33	18.27	25.46	-5.97	-66.46	-5.97	-66.46	1.013	0.36
**MR 9**	0.47 ± 0.24	17.76	145.82	16.10	33.74	-7.89	-77.59	-7.89	-77.59	1.027	0.31
**MR 10**	0.35 ± 0.06	58.14	277.75	-47.03	-89.69	33.46	30.39	13.49	47.46	1.005	0.37
